# Vitamin D Supplementation during Winter: Effects on Stress Resilience in a Randomized Control Trial

**DOI:** 10.3390/nu12113258

**Published:** 2020-10-24

**Authors:** Anita L. Hansen, Gina Ambroziak, David Thornton, James C. Mundt, Rachel E. Kahn, Lisbeth Dahl, Leif Waage, Daniel Kattenbraker, Pedro Araujo, Robert Murison, Knut Rypdal, Bjørn Grung

**Affiliations:** 1 Department of Psychosocial Science, University of Bergen, Christiesgt. 12, 5015 Bergen, Norway; 2 Centre for Research and Education in Forensic Psychiatry, Haukeland University Hospital, 5021 Bergen, Norway; lwaage@online.no (L.W.); knut.rypdal@helse-bergen.no (K.R.); 3 Sand Ridge Secure Treatment Center (SRSTC), P.O. Box 0700, 1111 North Road, Mauston, WI 53948, USA; Gina.Ambroziak@dhs.wisconsin.gov (G.A.); davidsmthornton@icloud.com (D.T.); James.Mundt@dhs.wisconsin.gov (J.C.M.); Rachel.Kahn@dhs.wisconsin.gov (R.E.K.); Daniel.Kattenbraker@dhs.wisconsin.gov (D.K.); 4 Forensic Assessment, Training and Research, LLC 1213 N. Sherman Avenue, Suite 334, Madison, WI 53704, USA; 5 Department of Seafood, Nutrition and Environmental State, Institute of Marine Research, P.O. Box 1870, Nordnes, 5817 Bergen, Norway; lisbeth.dahl@hi.no (L.D.); Pedro.Araujo@hi.no (P.A.); 6 Department of Biological and Medical Psychology, University of Bergen, Jonas Lies vei 91, 5021 Bergen, Norway; murison@uib.no; 7 Department of Chemistry, University of Bergen, Allégaten 41, 5007 Bergen, Norway; bjorn.grung@uib.no

**Keywords:** vitamin D, stress resilience, heart rate variability, heart rate, serotonin, cortisol

## Abstract

Vitamin D status may be important for stress resilience. This study investigated the effects of vitamin D supplements during winter on biological markers of stress resilience such as psychophysiological activity, serotonin, and cortisol in a placebo-controlled, randomized clinical trial. Eighty-six participants were randomly assigned to the Intervention (vitamin D) or Control (placebo) groups. Before and after the intervention participants were exposed to an experimental stress procedure. Psychophysiological activity was measured during three main conditions: baseline, stress, and recovery. Fasting blood samples were taken in the morning and saliva samples were collected at seven different time points across 24 h. Prior to intervention both groups had normal/sufficient vitamin D levels. Both groups showed a normal pattern of psychophysiological responses to the experimental stress procedure (i.e., increased psychophysiological responses from resting baseline to stress-condition, and decreased psychophysiological responses from stress-condition to recovery; all *p* < 0.009). Post-intervention, the Intervention group showed increased vitamin D levels (*p* < 0.001) and normal psychophysiological responses to the experimental stress procedure (*p* < 0.001). Importantly, the Control group demonstrated a classic nadir in vitamin D status post-intervention (spring) (*p* < 0.001) and did not show normal psychophysiological responses. Thus, physiologically the Control group showed a sustained stress response. No significant effects of vitamin D were found on serotonin and cortisol.

## 1. Introduction

Regular consumption of fatty fish has been shown to have beneficial health effects [1,2,3,4]. In a recent study a long-term fatty fish intervention improved resilience to stress in a group of forensic inpatients with complex mental health problems, while a diet without fatty fish impaired stress resilience [5]. Little is known about the specific nutrients and mechanisms of action underlying the beneficial effects of fatty fish consumption. The fatty fish study [5] was carried out during winter, and it was speculated that vitamin D may have played a key role in the results. Vitamin D has been shown to influence heart rate variability (HRV) [3], which is an important index of resilience [6]. Fatty fish are a rich source of vitamin D [7] and might prevent the frequently found decrease in vitamin D levels in humans during winter [8]. To better understand the role of vitamin D in relation to stress resilience, a robust randomized controlled trial (RCT) investigating the effects of vitamin D supplements is needed.

To investigate the effects of fatty fish consumption on resilience to stress, Hansen et al. [5] looked at psychophysiological responses to a mild cognitive stress procedure and psychophysiological recovery post-stress. Physiological recovery might be particularly important when it comes to stress resilience, as it illustrates a person’s ability to shut off the stress response after removal of a stressor [9]. What is striking in Hansen et al. [5], is that participants who ate fatty fish regularly throughout the winter were able to extinguish the physiological stress response after termination of the mild cognitive stress procedure. Participants in the control group receiving a diet without fatty fish were not. Participants in the control group showed sustained physiological arousal by continued suppressed HRV after the termination of the mild cognitive stress procedure [5]. The relationship between physiological recovery and vitamin D remains to be investigated.

The autonomic nervous system (ANS) is an important stress-response system, divided into the parasympathetic nervous system (PNS) and the sympathetic nervous system (SNS). Each system serves different functions. During stress, such as increased workload or threat, the SNS is activated to mobilize energy or fight or flight responses, if necessary. This causes secretion of neurotransmitters (e.g., norepinephrine and adrenaline) and increased heart rate (HR) producing shorter periods between successive heart beats [10]. The PNS is dominant when the body is relaxed and in a homeostatic balance [11]. The PNS is controlled by the vagus nerve, which mediates the PNS by acetylcholine. Due to this neurotransmitter, the PNS affects the HR much faster than the SNS (milliseconds instead of seconds). Consequently, the PNS causes longer inter-beat intervals [10]. HRV is regarded as an index of PNS activation [12].

Numerous studies have found that low resting HRV and high resting HR are associated with poor physical and mental health. Low resting HRV and a higher HR means that the body is constantly working overtime and constitutes a risk factor for mortality and morbidity [13,14]. Thus, variations in HR and HRV from a resting or safe condition to a stressful and challenging condition are a healthy, normal physiological response i.e., the system is homeostatically responsive [15]. Fiol-Veny et al. [16] investigated cardiovascular responses to a procedure known to elicit higher stress responses (i.e., the Trier Social Stress Test). They found that HR increased from baseline to stress and it decreased from stress to recovery. HRV decreased from baseline to stress and it increased from the stress condition to recovery. To investigate the effects of vitamin D on resilience to stress it is important to examine physiological responses to an experimental design consisting of different conditions, such as a resting baseline, stressful conditions, and resting recovery.

HRV is a non-invasive measure, and it is not known whether it is affected directly or indirectly by fatty fish consumption. There is speculation that serotonin could be a mechanism of action in this regard [3,4], based on the evidence linking vitamin D to serotonin regulation [17]. Serotonin regulation is involved in a range of both physiological (e.g., energy balance, sleep, and arousal, including activation of parasympathetic outflow or regulation of HRV) [18,19,20] and psychological functions influencing emotions and behavior [21]. In the Hansen et al. study [4], the control group, receiving a diet without fatty fish, showed increased HR from summer to winter. Thus, it was speculated that the ANS might be sensitive to seasonal changes associated with the fluctuations in vitamin D status. Vitamin D status has its nadir in April as a consequence of the absence of sufficient sun exposure, especially in the Northern hemisphere [22,23]. Investigating the effects of vitamin D supplementation throughout the winter on serotonin levels might provide insights in this regard.

There are seasonal variations in serotonin, with higher levels of serotonin occurring during the winter compared to spring and summer. Importantly, subjects with externalizing behavior problems have been shown to have greater seasonal fluctuation compared to normal healthy controls [24]. More knowledge about the relationship between vitamin D and serotonin in patients with severe mental health problems, such as antisocial behavior problems, is of particular importance since antisocial behavior problems are associated with serotonin system dysfunction [25]. Despite considerable research investigating the relationship between serotonin and severe mental health problems, the direction of this dysfunction (i.e., high or low levels of serotonin) remains a matter of debate [25]. The inconsistencies in the literature may be related to the seasonal variation in serotonin. A double-blinded, repeated-measures study design investigating the effects of a vitamin D intervention throughout winter could be of particular importance in addressing this issue.

Another important biomarker of stress is cortisol. In normal healthy participants cortisol levels are inversely related to HRV [26]. Forensic inpatients with complex mental health disorders, such as antisocial behavior problems, have high rates of early traumatic stress, such as childhood trauma [27,28]. Most research investigating the relationship between early adversity and cortisol responses to stress (e.g., public speaking and mental arithmetic) in adults shows blunted cortisol response [see 29 and 30 for an overview]. When it comes to the relationship between early adversity and diurnal hypothalamic-pituitary-adrenal (HPA) regulation in adulthood, the literature largely suggests that adversity does not affect diurnal cortisol cycles [29,30]. However, Brewer-Smyth and Burges [31] found that females exposed to early sexual abuse had decreased diurnal cortisol secretion [see 29 for an overview]. Additionally, maltreated children exhibited a flattened diurnal cortisol response, which returned to normal after a family based treatment program [32].

To assess abnormalities in cortisol responses it is important to look at cortisol awakening responses (CAR), the rise in cortisol from awakening to about 30 min after awakening [30]. Normally there is a 75% increase in cortisol level from awakening to 30 min after awakening, followed by a gradual decrease during the day [33]. Low morning cortisol predicts antisocial behavior in adolescents [34]. A blunted CAR response, together with depression, has been associated with violent behavior as well [35]. Unfortunately, few studies investigating antisocial behavior have measured CAR [30].

Cortisol responses to stress have important behavioral implications, and blunted cortisol responses to stress are associated with poor emotional and behavioral regulation [15]. Cortisol hypo-reactivity may predict poor treatment outcomes [30]. The functioning of the CAR is not clearly understood, but it has been hypothesized to be important for mobilization of energy to address demands of the upcoming day [33]. Thus, identification of strategies that improve cortisol responses might also improve mobilization of energy and stress resilience. Dietary interventions may be promising strategies in this respect [36]. However, research studying this relationship between dietary interventions and cortisol is still rare and needs to be explored.

Overall, studies of the effects of fatty fish consumption [3,4,5] raised new questions regarding the role of important nutrients such as vitamin D and its effect on mechanisms of stress resilience such as HR and HRV recovery, possible mechanisms of action involved such as serotonin, as well as possible effects on other mechanisms of stress resilience such as cortisol responses. Moreover, in Hansen et al. [5] participants were exposed to a mild cognitive stress procedure. To extend this research further investigation has to focus on an enhanced stress procedure.

Thus, the primary aim of the present study was to investigate the effects of vitamin D supplements throughout winter in relation to an enhanced stress procedure. We expected that vitamin D supplementation through winter would prevent a nadir in vitamin D level during spring. In addition, we expected that the nutritional supplements would contribute to the maintenance of healthier patterns of psychophysiological responses to an enhanced stress procedure (i.e., decreased HRV and increased HR from baseline to stress conditions, and increased HRV and decreased HR from stress conditions to recovery) [5,16]. Along with a seasonal reduction in vitamin D status we expected the Control group would show a pattern of sustained physiological arousal during exposure to the experimental stress procedure (i.e., no changes from baseline to stress and no changes from stress conditions to recovery) [5].

To gain more knowledge about the mechanisms of action, a secondary aim of this study was to investigate the effects of vitamin D in relation to serotonin and cortisol responses. Based on the relationship between vitamin D and serotonin [17], seasonal fluctuations of serotonin levels, and evidence that people with externalizing behavior problems show amplified rhythms compared to normal healthy controls [24], it was expected that those receiving vitamin D supplements during winter would show a reduction in the typical seasonal decrease in serotonin. Given the lack of studies investigating the effects of vitamin D supplements on cortisol responses in participants with severe mental health issues, we also sought to address this gap in the literature.

## 2. Materials and Methods

### 2.1. Study Design

This study is a parallel randomized double-blind placebo controlled trial investigating the effects of vitamin D supplements on mechanisms of stress resilience in forensic inpatients. The study was a collaboration among Norwegian and US researchers. As such, the study protocol was reviewed by the Regional Committee for Medical Research Ethics, Western Norway (REK-West; 2017/1520; 6 October 2017), as well as the Sand Ridge Secure Treatment Center Institutional Review Board (IRB00002675; FWA00021540; 7 August 2017). We have followed the updated Consolidated Standards of Reporting Trials (CONSORT) criteria and the trial is registered at ClinicalTrials.gov (Identifier: NCT03336125).

### 2.2. Sample

Sample size was based on an a priori power analysis performed based on published data comparing vitamin D in human serum from supplemented and non-supplemented populations [37]. The *a priori* power analysis (α = 0.05) indicated that 50 patients (i.e., number of participants; *n*) per group was needed for a Type II error level of 20%.

**Figure 1:**
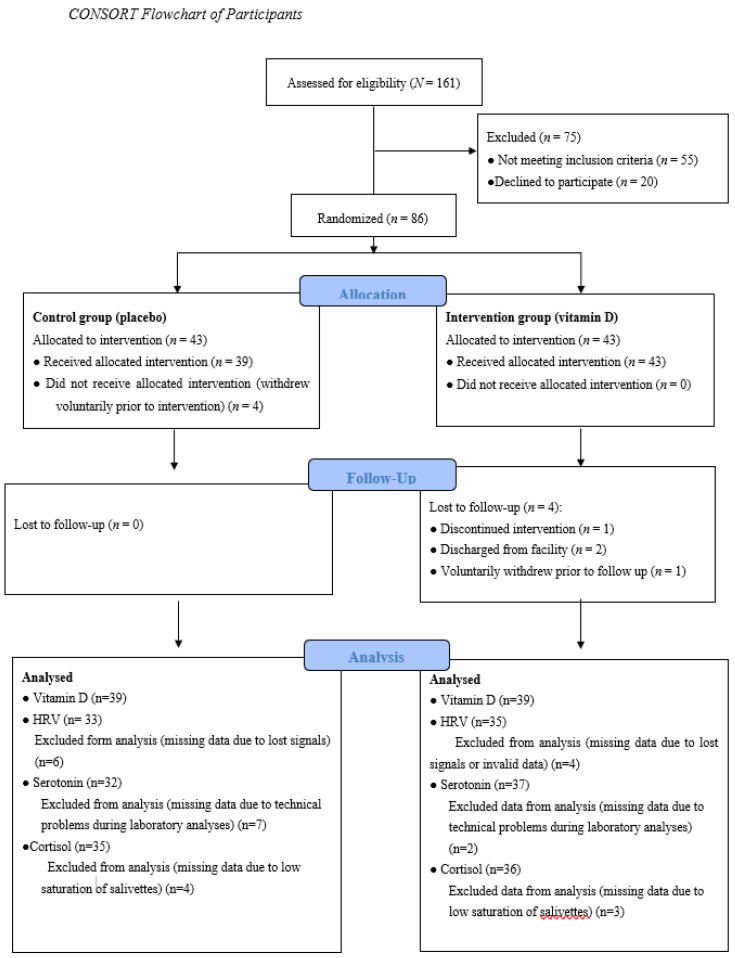
The study progress. HRV = Heart Rate Variability.

All patients at a secure inpatient treatment facility were invited to participate in a research study on key nutrients and mental health, and 161 male participants were assessed for eligibility. Seventy-five were excluded after initial screening, 20 declined to participate, and 55 did not meet inclusion criteria. Patients were excluded if they were already taking vitamin D supplements (*n* = 29), had an IQ <70 (*n* = 16), had a severe mental illness such as schizophrenia and schizoaffective disorder (*n* = 5), had a major neurocognitive disorder, neurodevelopmental disorder, or history of traumatic brain injury (*n* = 2), or were unable to complete the protocol (i.e., did not speak fluent English (*n* = 1); tremor (*n* = 1); legally blind (*n* = 1)). Thus, the eligible pool of participants was limited to a total of 86 volunteers who were randomized into two groups, Control group (placebo) or Intervention group (vitamin D) at study entry. Each group consisted of 43 participants. Figure 1 presents a flow diagram of the study progress.

Descriptive characteristics of the sample are presented in Table 1. All participants in this study were patients detained or civilly committed as sexually violent persons in an inpatient treatment facility in the US. Participants ranged in age from 31 to 81 years (M = 48, standard deviation (SD) = 11). The majority of the sample was White (72%) followed by Black (19%), Native American (8%), Asian/Pacific Islander (1%). Personality disorders (e.g., antisocial and borderline personality disorders) and substance use disorders were common in both participant groups (see Table 1). Other diagnoses, such as mood or trauma-related disorders, were less common. However, scores on trauma questionnaires, i.e., Childhood Trauma Questionnaire-Short Form (CTQ-SF), and the Impact of Event Scale-Revised (IES-R) indicated that childhood traumas and post-traumatic stress symptoms were common in both groups. Importantly, about 50% of the total sample had a score above the cut off score on the IES-R (i.e., over 24), which means that post-traumatic stress disorder (PTSD) is a clinical concern (see Table 1).

Before randomization, participants were matched in Norway on age and IQ. Each participant was assigned a participant number. The Mersenne twister random number generator in MATLAB (MathWorks, Natick, MA, USA) with the current time as seed was used to assign each of the matched pairs. External personnel were used to assign vitamin D or placebo to the two randomized groups. This information was not available to either the US or the Norwegian teams. The random allocations to the groups were completed before all participants were enrolled and had completed baseline testing (pre-intervention battery). Thus, all researchers, staff and participants were blinded.

Participants were recruited (recruitment began 12 October 2017) by oral and written information about the study. Before the start of the experiment, subjects were informed of their rights, including the right to withdraw from the study at any time for any reason, and signed an informed consent statement. Participation in this study did not have any positive or negative consequences with regard to their confinement or the services received at the treatment facility.

### 2.3. Intervention

The Intervention group received vitamin D pearls (40 µg cholecalciferol corresponding to 1600 IU), while the Control group received placebo pearls (120 mg olive oil). These were provided as identical clear round soft gelatin pearls containing a light brown oil. Participants received the supplements daily from 7 January 2018 to 22 May 2018. The supplements were delivered to the participants by health services staff at the institution and along with their daily medication. Compliance with taking supplements was tracked by recording a “1” if the participants took the capsules and 0 if the participant did not take the capsules. If the participant had to leave the institution for some reason (e.g., out to medical appointments or return to court) the supplements were sent along with his other medication to be administered off-site. The present study had a high degree of compliance (about 97%).

The intake of vitamin D was based on recommendations from the National Institute of Health (NIH). The current American and Nordic upper limit for vitamin D intake is 100 µg/day (4000 IU/day). This is the highest level of daily intake that is likely to pose no risk of adverse health effects to almost all individuals in the general population [39]. The vitamin D pearls in the present study contribute 38% of the upper limit intake level. The pearls were Halal- and Kosher-certified and produced by Pharma Nord, Denmark.

Participants were offered up to a $40 compensation for study participation. Providing compensation to voluntary participants is a common practice in the US. Participants were paid $10 for completing the pre-intervention test battery. Both groups were required to maintain compliance with the supplement (or placebo) regimen throughout the study. Participants who missed more than three doses of supplements during any week of the intervention period were no longer eligible to continue in the study. Participants maintaining regimen compliance and completing the post-intervention test battery were compensated with an additional $30 upon study completion.

### 2.4. Measures

#### 2.4.1. Psychophysiological Measures

Psychophysiological responses (HR and HRV) before, during, and after the experimental stress test battery were registered by the Actiheart System (Cambridge Neurotechnology Ltd., Cambridge, UK) [40], a compact lightweight device that records heart rate and variability of R-R inter-beat intervals (IBI). The Actiheart clips onto a single electrocardiogram (ECG) electrode (M-00-S/50 Blue Sensor) with a short ECG lead to another electrode that detects the ECG signal. The Actiheart was placed on the upper chest.

Artifacts in IBI were screened for and handled manually in the Actiheart program.

HR was measured as the average heart rate in beats per minutes for the analysis epoch (1 min). HRV was measured as high frequency (HF) power (0.15–0.4 Hz) derived using fast Fourier transform (FFT). These calculations were performed in the Actiheart program. Moreover, analyzed raw data were exported to Excel for further processing. For each participant we had a detailed time log indicating the exact start and end time for each test condition (i.e., baseline, each cognitive test and recovery). Based on this time log an average HR and HF-HRV for each condition was calculated.

#### 2.4.2. Self-Report Questionnaires

The Childhood Trauma Questionnaire-Short Form (CTQ-SF) [41,42] was used to assess early childhood stressors associated with different types of trauma. The CTQ-SF is a self-report questionnaire consisting of 28 items measuring five types of maltreatment: Physical Abuse, Emotional Abuse, Sexual Abuse, Physical Neglect and Emotional Neglect. In the present study Cronbach’s alpha for the five subscales were 0.89, 0.88, 0.91, 0.69, and 0.91.

The Impact of Event Scale-Revised (IES-R) [43] was included as a measure of post-traumatic stress symptoms. The IES-R has 22 items, divided into three symptom subscales; Intrusion, Avoidance and Hyperarousal. Our data showed that the Cronbach’s alpha for the three subscales were 0.89, 0.79, and 0.85, respectively.

#### 2.4.3. Blood Sampling

Fasting blood was collected from participants in the morning between 06:30 and 09:30 by biomedical health services staff at the treatment facility pre and post intervention. Venous blood for serum preparation was collected in BD Vacutainer^®^ vials and set to coagulate for minimum 30 min before centrifuging (10:00–13:00 G, 20 °C, 10 min) within 60 min. The samples were transported the same day to a routine clinical laboratory near the study site (ACL laboratories, Milwaukee, WI, USA) for analysis of vitamin D (i.e., 25(OH)D) and serotonin. For the determination of vitamin D a competitive immunoassay was used and a vitamin D analog labeled with fluorescein for detection. The unit of measurement for 25(OH)D was nanomole per liter (nmol/L). For the determination of serotonin, a quantitative high-performance liquid chromatography (HPLC) method was used. The unit measurement for serotonin was nanograms per milliliter (ng/mL).

#### 2.4.4. Saliva Sampling

The Salivette collection device was used to collect saliva samples. The Salivary cortisol was run using the Salimetrics^®^ Cortisol Enzyme Immunoassay Kit. It is a competitive immunoassay specifically designed and validated for the quantitative measurement of salivary cortisol.

### 2.5. Data Collection and Experimental Stress Procedure

The present study is part of a larger project investigating the effects of vitamin D on mental health and resilience to stress. Participants were exposed to an extensive battery of tests before and after the intervention. Pre-intervention testing started 16 November 2017 and ended 29 December 2017. Post-intervention testing started after about 4 months with supplements, i.e., 2 April 2018 and ended 22 May 2018. Thus, the post-intervention testing took place while the participants were still taking supplements. Testing procedures included experimental cognitive performance tasks, physiological measures, questionnaires (concerning mental health, trauma and resilience), sleep registration, and laboratory measures (saliva, blood and urine samples). With the exception of the laboratory measures (see description in Measures), all measures of the test battery were collected in a single pre-intervention and a single post-intervention testing session.

Computer-based cognitive tasks used in this experiment are well known, such as the Tower of London (ToL) e.g., [44], Tower of Hanoi (ToH) e.g., [45], Iowa Gambling Task (IGT) e.g., [46] and an N-back task e.g., [47]. All tasks were administered in the E-prime system (Psychology Software Tools, Pittsburgh, PA). Participants completed the cognitive tasks in randomized order. In each of the experimental tasks, reaction time and accuracy data were registered by the computer. The participants were instructed to respond as quickly and accurately as possible. Exposure to cognitive experiments like this elicit physiological stress responses and can be regarded as mild stress inducing procedures e.g., [5,48,49]. To increase the stress from the procedures previously used in Hansen et al. [5], participants in the present study were exposed to aversive noise during the performance of the cognitive tasks. To obtain noise that varied in intensity and frequency, an excerpt of mass spectrometry data of a fillet of salmon was converted to a WAV file using the function ‘audiowrite’ in MATLAB (The MathWorks, Natick, MA, USA). The resulting noise was delivered through headphones during the performance of the cognitive tasks. The file can be found in the Supplementary material.

Distracting noise is a common stimulus used in laboratory studies to elicit stress responses [50]. Such noise should be experienced as uncomfortable, but not physically painful. Thus, ethical considerations allowed each participant to select a noise level between 80 and 100 dB that they found to be “annoying, but not painful” before the start of the experimental sessions (see Table 1 for descriptive information about volume of noise). The mean volume of the noise for the Control and Intervention groups during the pre-intervention tests was 92.05 (SD = 8.94) and 93. 85 (SD = 7.58) dB, respectively (*p* < 0.35). At post-intervention testing the mean noise levels were 94.21 (SD = 7.58) and 94.36 (SD = 7.88) dB, respectively (*p* < 0.93).

Psychophysiological activity was registered during a resting baseline for five minutes before presentation of the cognitive tasks, during completion of the cognitive tasks (stress-condition), and during a resting recovery for five minutes after finishing the cognitive tasks. The experimental procedures required about 60–90 min to complete, and all participants were tested individually.

**Figure 2:**
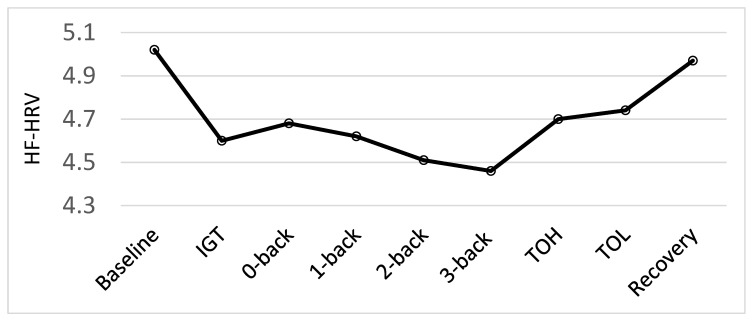
Psychophysiological responses (High-frequency heart rate variability; HF-HRV) during each condition: baseline, Iowa Gambling Tasks (IGT), N-back tasks (0-back, 1-back, 2-back and 3-back), Tower of Hanoi (ToH), Tower of London (ToL) and recovery, before the intervention for all participants.

This study was designed to extend and increase the stress levels experienced (and recovered from) in comparison to the procedures used in Hansen et al. [5] by adding aversive noise during completion of the cognitive testing procedures. To test whether the present stress procedure expanded the stress procedure used in Hansen et al. [5], effects of the different conditions (baseline, cognitive tests and recovery) on HF-HRV and HR were tested by a repeated measure of analysis of variance (ANOVA) prior to analyses of the effects of the vitamin D intervention. Looking at the physiological responses for the whole sample before the intervention the results revealed a main effect of conditions for HF-HRV, F (8, 552) = 11.87, *p* = 0.001. As illustrated in Figure 2, there was a significant decrease in HF-HRV from baseline to all test conditions, as well as a significant increase in HF-HRV from all test-conditions to recovery (all *p* < 0.002). Moreover, the results demonstrated the sensitivity of the HF-HRV as a measure of cognitive stress [5,49]. There was a significant decrease from the easy non-executive functioning task, the 0-back, to the more difficult 2-back (*p* = 0.023) and 3-back (*p* = 0.004) tasks. There was also a significant decrease from the easier 1-back task to the difficult 3-back task (*p* = 0.045). Additionally, there was a significant increase from the 3-back task to the ToH (*p* = 0.003) and ToL (*p* < 0.001). However, as there was a significant decrease in HF-HRV from baseline to all the tests and a significant increase in HF-HRV from all the tests to recovery, our stress regimen was successful. The current study aimed to examine biological markers of resilience to stress using an enhanced stress procedure and not the psychophysiological response to each test specifically. To test the hypotheses related to this particular study, therefore, we established an HF-HRV average score for the stress conditions based on physiological activity to all the cognitive test conditions. The same procedure was followed for HR data.

Diurnal cortisol pattern and cortisol responses to stress were measured by cortisol samples collected seven times across the 24 h of the pre-intervention and post-intervention experimental testing days. Cortisol saliva was collected the evening before the experimental testing session (between 7:00 p.m. and 9:00 p.m.; T1), upon waking the morning of the experimental session (T2), 15 min after waking up (T3), 30 min after waking up (T4), 15 min before the start of the experimental stress testing procedures (T5), 15 min after completing the stress testing procedures (T6) and a final sample the evening (between 7:00 p.m. and 9:00 p.m.) of the experimental testing day (T7). Thus, cortisol samples T1, T2, T3, T4 and T7 were used to measure diurnal cortisol patterns, including the CAR (i.e., T2, T3 and T4). Cortisol sample T5 and T6 were used to measure cortisol responses to stress. All participants were instructed to rinse their mouth with water about 10 min before the test, but not to eat, drink and brush their teeth during the 60 min prior to each saliva test.

### 2.6. Statistical Analyses

In order to investigate the effects of vitamin D vs. placebo in relation to psychophysiological responses (HF-HRV and HR) to an experimental stress procedure, a 2 × 2 × 3 mixed-model ANOVA design was used with one between-subject and two within-subjects factors. Intervention vs. Control group was the between-subjects factor, and the pre- vs. post-intervention testing by baseline, stress, and recovery periods were the within-subjects factors. Cortisol responses were likewise analyzed by a 2 × 2 × 7 mixed-model ANOVA with Intervention vs. Control as a between-subjects factor, and pre- vs. post-intervention testing with seven observation times (T1, T2, T3, T4, T5, T6, and T7) as within-subject factors. Changes in serotonin and vitamin D from pre- to post-intervention were analyzed by a two-way between-subjects ANOVA. Our specific hypotheses related to psychophysiological responses and serotonin, based on previous investigations [5,16,24] respectively, were tested regardless of significant omnibus F-tests [51] using adjusted Bonferroni probability values following Simes’ procedure [52] to control for Type 1 error. Eight pre-planned comparisons included baseline to stress and from stress to recovery for both groups at both pre- and post-intervention for psychophysiological responses, and at comparison of serotonin before and after the intervention was completed. To investigate the magnitude of the significant differences between means, effect sizes were calculated as Cohen’s d [53].

Relationships among the different variables were also explored by Pearson product-moment correlation analyses. To explore the relationship between biological markers of stress resilience zero-order correlation analyses were used. Concerning the relationship between vitamin D and biological markers of stress resilience we also performed partial correlations controlling for age with regard to the cardiovascular responses as it has been argued that age may influence HRV [54].

Prior to data analyses HRV, serotonin and cortisol values were log transformed in order to normalize the distribution.

## 3. Results

### 3.1. Descriptive Statistics

Means and standard deviations for all variables are presented in Table 2. These are descriptive variables such as age, IQ, body mass index (BMI), scores on CTQ-SF and IES-R, as well as medication (antidepressant and cardiovascular) for each group at pre-intervention. To look for differences between the groups in medication, we counted prescribed medication for each participant (i.e., antidepressants and cardiovascular medication). The number of prescribed medications ranged from 0 to 5. Moreover, Table 2 presents means and standard deviations for all the dependent variables pre- and post-intervention for both groups.

### 3.2. Effects of Intervention on Vitamin D Status

Analysis of variance showed a main effect of groups, F (1,76) = 18.93, *p* < 0.001, η2 = 0.20, showing a significantly higher level of vitamin D in the Intervention group (*p* < 0.001) compared to the Control (placebo) group (*p* < 0.001; d = 0.88). Moreover, there was a significant interaction between groups and time (pre- vs. post-intervention), F (1,76) = 37.50, *p* < 0.001, η2 = 0.33. Follow-up tests showed a significant increase in vitamin D from pre- to post-intervention in the Intervention group, (*p* < 0.001; d = 0.69), while the Control group showed a significant decrease in vitamin D from pre- to post-intervention (*p* < 0.001; d = 0.60). Additionally, there was a significant difference between the groups in vitamin D status post-intervention (*p* < 0.001; d = 1.56).

### 3.3. Effects of Intervention on Biological Mechanisms of Stress Resilience

#### 3.3.1. Psychophysiological Measures

HF-HRV: repeated measures ANOVA showed a significant main effect of the experimental stress procedure (i.e., baseline-stress-recovery) F (2, 132) = 16.84, *p* < 0.001, η2 = 0.20. Follow-up tests revealed that overall (both groups at pre- and post-intervention pooled together) there was a significant decrease in HF-HRV from baseline to stress (*p* < 0.001; d = 0.21) and an increase from stress to recovery (*p* < 0.001; d = 0.23). There was also a significant interaction between conditions and groups, F (2, 132) = 4.631, *p* = 0.011, η2 = 0.07. Follow-up tests showed the Intervention group had a significant decrease in HF-HRV from baseline to stress (*p* = 0.002; d = 0.19) and a significant increase from stress to recovery (*p* < 0.001; d = 0.33). The Control group also had a significant decrease in HF-HRV from baseline to stress (*p* < 0.001; d = 0.23), but did not show a statistically significant increase from stress to recovery (*p* = 0.053; d = 0.12).

**Figure 3:**
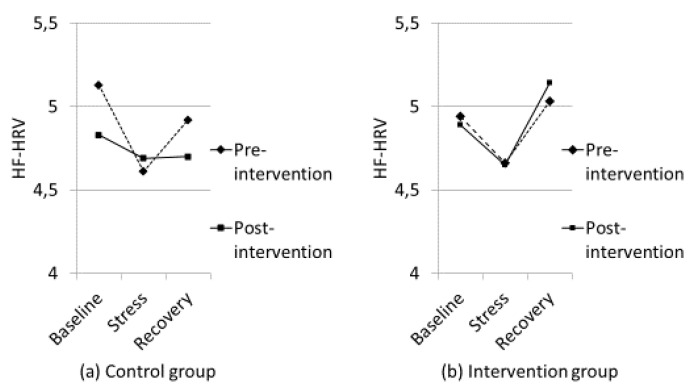
Psychophysiological responses (High-frequency Heart Rate Variability; HF-HRV) to baseline, stress (the average score for all cognitive tasks) and recovery for the Control group (**a**) and the Intervention group (**b**).

The interaction among time (pre- and post-intervention), experimental stress procedure, and groups was not significant, F (2, 132) = 2.116, *p* = 0.125, η2 = 0.03. However, for the Intervention group-adjusted Bonferroni demonstrated a significant decrease from baseline to stress (*p* = 0.009; d = 0.21) and a significant increase from stress to recovery at pre-test (*p* < 0.001; d = 0.29). A generally identical pattern of results was found post-intervention. From baseline to stress there was a decrease in HF-HRV, but this was not statistically significant according to the adjusted Bonferroni (*p* = 0.036; d = 0.18) (Adj. Bonferroni *p* = 0.013). However, the increase from stress to recovery was statistically significant according to the adjusted Bonferroni (*p* < 0.001; d = 0.38) (see Figure 3). Moreover, adjusted Bonferroni showed that the Control group had a significant decrease in HF-HRV from baseline to stress (*p* < 0.001; d = 0.35) and a significant increase from stress to recovery (*p* = 0.005; d = 0.22) pre-intervention. However, the results post-intervention were different for the Control group. There was no significant change from baseline to stress (*p* = 0.208; d = 0.11) or from stress to recovery (*p* = 0.90; d = 0.00) (see Figure 3).

HR: there was a significant main effect of the experimental stress procedure, F (1,66) = 20.69, *p* = 0.001, η2 = 0.24. Overall, there was a significant increase in HR from baseline to stress (*p* < 0.001; d = 0.13), and a significant decrease from stress to recovery (*p* < 0.001; d = 0.20).

The interaction among time, groups and experimental stress procedure was not significant, F (2, 132) = 0.455, p = 0.636; η2 = 0.007. However, adjusted Bonferroni tests showed the Intervention group had a significant increase in HR from baseline to stress (*p* = 0.003; d = 0.18), and a significant decrease from stress to recovery (*p* < 0.001; d = 0.33). The same pattern of results was observed post-intervention for this group. There was a significant HR increase from baseline to stress (*p* = 0.005; d = 0.13), and a significant decrease from stress to recovery (*p* < 0.001; d = 0.22). The Control group showed the same pattern as the Intervention group at pre-intervention, i.e., a significant increase in HR from baseline to stress (*p* = 0.006; d = 0.13) and a significant decrease from stress to recovery (*p* = 0.001; d = 0.18). However, at post-intervention the Control group did not show significant changes (*p* = 0.100; d = 0.09 and *p* = 0.117; d = 0.08, respectively).

#### 3.3.2. Serotonin

Analysis of the serotonin showed no significant effect of groups F (1,67) = 0.78, *p* = 0.381, η2 = 0.01. However, a significant main effect of time, F (1,67) = 9.92, *p* = 0.002, η2 = 0.13, revealed a significant reduction in serotonin from pre- to post-intervention, i.e., from winter to spring (*p* = 0.002; d = 0.30). The interaction between time and groups was not significant F (1,67) = 0.026, *p* = 0.87, η2 = 0.00. However, adjusted Bonferroni tests revealed that both the Control group (*p* = 0.045; d = 0.28) and the Intervention group (*p* = 0.018; d = 0.31) had a significant decrease in serotonin from winter to spring.

#### 3.3.3. Cortisol

**Figure 4:**
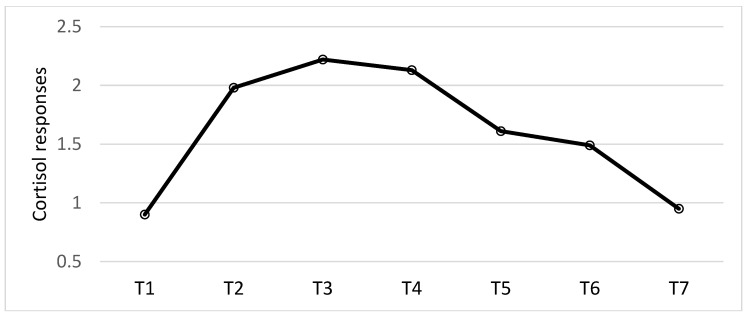
The diurnal cortisol responses (log transformed), including the cortisol awakening responses (CAR, T2, T3 and T4) and the stress responses (T5 and T6). Raw cortisol responses are reported in Table 2.

Analyses of cortisol responses revealed a main effect of the time of day, F (6, 414) = 99.97, *p* < 0.001; η2 = 0.59. Follow-up tests revealed there was a significant increase from the evening prior to the experimental testing sessions (T1) to waking up on the testing day (T2) (*p* < 0.001; d = -1.64). From waking up (T2) to 15 min after wakeup (T3) there was a peak of cortisol (*p* = 0.001; d = 0.32). From wake up (T2) to 30 min after waking up (T4) the cortisol response was still higher than at wake up, but this was not significant (*p* = 0.057; d = 0.20) (T2, T3 and T4 = CAR). Thus, there was a slight, but non-significant, decline from T3 to T4 (*p* = 0.22; d = 0.13). There was a significant reduction from 30 min after waking up (T4) to 15 min before the start of the experimental stress procedure (T5; *p* < 0.001; d = 0.81), but not from pre-stress to 15 min post-stress (T6) (*p* = 0.11; d = 0.21). There was a significant decrease from post-stress (T6) to the evening of the experimental testing day (T7) (*p* < 0.001; d = 0.81; see Figure 4). No other results were significant.

### 3.4. Correlations

#### 3.4.1. Correlations between the Different Biological Markers of Stress Resilience

Cardiovascular responses and cortisol: Pre-intervention no relationships between cardiovascular responses and cortisol responses were observed (all r < −0.19, all *p* > 0.105). However, post-intervention HF-HRV baseline correlated positively with T2 (r = 0.27, *p* = 0.023), T3 (r = 0.27, *p* = 0.020) and T4 (r = 0.24, *p* = 0.046). Moreover, HF-HRV recovery correlated positively with T3 (r = 0.23, *p* = 0.049). In addition there were some marginal relationships between HF-HRV recovery and T4 (r = 0.22, *p* = 0.061) and T5 (r = 0.22, *p* = 0.068). Pre-intervention no significant relationships between HR and cortisol responses were observed (all r < −0.14, all *p* > 0.257). However, post-intervention a negative relationship between HR-baseline and T5 (r = −0.28, *p* = 0.019) was observed. Furthermore, there was a negative relationship between HR-stress and T5 (r = −0.29, p = 0.017) and HR-recovery and T5 (r = −0.28, *p* = 0.017).

Cardiovascular responses and serotonin: Pre-intervention there were significant correlations between HF-HRV and serotonin. This was true for all conditions, i.e., baseline (r = 0.44, *p* < 0.001), stress (r = 0.40, *p* = 0.001) and recovery (r = 0.40., *p* < 0.001). The same pattern of results was found at post-intervention (r = 0.36, *p* = 0.002), (r = 0.30, *p* = 0.009) and (r = 0.41, *p* < 0.001), respectively. No significant relationships were found for HR and serotonin (all r < −0.19, all *p* > 0.129).

Serotonin and cortisol: Pre-intervention, a negative relationship between serotonin and T1 was observed (r = 0.24, *p* = 0.047). In addition a weak, but non-significant relationship was observed for serotonin and T7 (r = 0.21, *p* = 0.077). No significant relationships were observed post-intervention (all r < −0.10, all *p* > 0.385).

#### 3.4.2. Correlations between Vitamin D and Biological Markers of Stress Resilience

Zero-order correlations showed significant relationships between vitamin D and serotonin both pre- and post-intervention, (r = 0.27, *p* = 0.025) and (r = 0.23, *p* = 0.046), respectively. No other significant zero-order correlations were found using a significance level of 0.05 (see Table 3).

However, a closer look at the partial correlations demonstrated an interesting change in the strength of the relationship between vitamin D and HF-HRV recovery from pre- to post-intervention, i.e., (r = 0.03, *p* = 0.810) at pre-intervention compared to (r = 0.21, *p* = 0.067) at post-intervention (see Table 3).

## 4. Discussion

The current study aimed to investigate the effects of vitamin D supplements on markers of stress resilience in male forensic inpatients. At study entry the groups did not differ in vitamin D status. The Intervention group showed a significant increase while the Control group showed a significant decrease in vitamin D status following the intervention, producing a significant difference between the groups by the end of the intervention period. Pre-intervention the two groups also showed the same pattern of psychophysiological responses to the experimental test procedure, i.e., significant changes from baseline to stress, as well as changes from stress to recovery. This was true for both HF-HRV and HR. The Intervention group showed this pattern of results post-intervention as well. However, the Control group did not show significant variations in HF-HRV nor HR post-intervention. Moreover, both groups showed a significant decrease in serotonin during wintertime. No effects of the vitamin D intervention on serotonin or cortisol were found.

Analysis of the vitamin D levels showed both groups had a vitamin D status just below the U.S. recommended level of 75 nmol/L [55] at study entry (November/December 2018). The Control and the Intervention groups had mean levels of 59 (SD = 21) and 63 (SD = 16) nmol/L, respectively. Thus, at the beginning of the study both groups had normal to sufficient vitamin D levels; a level between 51–74 nmol/L is regarded as normal/sufficient [56]. However, by spring the Control group showed the classic nadir in vitamin D status (M = 47, SD = 16) while the Intervention group reached the U.S. recommended level (M = 76, SD = 21 nmol/L). Thus, the vitamin D supplementation intervention throughout winter prevented the classic nadir in vitamin D status in spring for the Intervention group and raised the concentration to recommended levels.

This investigation of the effects of vitamin D supplementation through winter months on stress resilience supports and extends earlier results [5]. Support is provided by the fact that both studies used nutritional interventions (i.e., fatty fish consumption [5] and vitamin D supplementation) and found a common pattern of psychophysiological responses to stress-inducing experimental procedures at both pre- and post-intervention. The Control groups in both studies revealed the same pattern as the Intervention groups prior to the nutritional intervention, but they showed a different pattern post-intervention, reflecting sustained physiological arousal following exposure to the stress-inducing test procedures.

The present study extends the previous investigation [5] because it used an enhanced stress procedure by adding aversive noise and intervening with vitamin D supplements for only three to four months, rather than nutritional intervention with fatty fish for six months. The significant changes in physiological responses (both HF-HRV and HR) from baseline to stress found in both groups prior to the nutritional intervention in the present study confirmed the stress inducing procedures were significantly greater than the experimental procedures used by Hansen et al. [5]. The stress procedure in Hansen et al. [5] can be regarded as a mild stress-inducing procedure because there was no change in HF-HRV from resting baseline to the mild stress-condition in that study, but the increase in HF-HRV from the mild stress- to recovery period was significant in both groups prior to the nutritional intervention. After the intervention, the Control group showed an impaired HF-HRV-recovery (i.e., sustained arousal after termination of the mild stress). Importantly, the present study demonstrated that physiologically, the Control group was in a constant state of stress by the end of the intervention period as there was no changes in HF-HRV or HR from baseline to stress or from stress to recovery. Thus, for the Control group the characteristic “V” pattern of HF-HRV (and inverse V for HR) found in both groups pre-intervention was transformed to a flat “–” pattern by the end of the intervention (see Figure 3). There were no significant correlations between vitamin D and cardiovascular responses. However, based on the fact that the groups did not differ in vitamin D status at pre-intervention, but there was a significant difference between the groups at post-intervention, it is worth being aware that the correlation between vitamin D and HF-HRV recovery at pre-intervention was r = 0.03, but it was r = 0.21 at post-intervention when controlling for age (see Table 3).

As shown in Figure 3, the Intervention group in this study demonstrated the characteristic V pattern of HF-HRV both before and after the nutritional intervention. It should be noted that the reduction in HF-HRV from baseline to stress post-intervention for the Intervention group did not reach a statistically significant level after the adjusted Bonferroni procedure (*p* = 0.036 and adjusted Bonferroni *p* = 0.013). It is important to note that the participants had been exposed to the aversive noise during pre-intervention testing, so the noise may have been anticipated and not as novel during post-intervention testing. The Intervention group still showed a significant increase in HF-HRV from stress to recovery post intervention, however, illustrating a termination of the stress response. Importantly, the HR results demonstrated a significant increase in HR from baseline to stress, and a significant reduction in HR from stress to recovery.

The present study also aimed to investigate the effects of vitamin D on serotonin, as an association between vitamin D and serotonin has been previously found [17]. A significant relationship between serotonin and vitamin D was also observed in the current study, both pre- and post-intervention. As people with externalizing behavior problems have shown amplified seasonal variations in serotonin [24], it was hypothesized that the Intervention group would show a weaker seasonal decrease in serotonin. However, this was not found. Both groups showed the same significant decrease from pre- to post-intervention. The present study investigated a seemingly homogeneous group as all participants were forensic inpatients, characterized by severe mental health problems, and they were all from the same institution. As has been argued before, severe mental illnesses are characterized by a relatively high degree of heterogeneity as well [25]. Thus, the relationship between vitamin D and serotonin might not be straightforward. To gain more knowledge about serotonin as a possible mechanism of action more research is needed.

Looking at the cortisol results, there was no effect of vitamin D on cortisol responses, neither in the diurnal pattern including the CAR nor the responses to stress. The cortisol results overall (main effect of sample collection time, both groups pooled together) showed the classic diurnal cortisol pattern, with higher cortisol response in the morning compared to the evening. However, the morning cortisol responses (i.e., wake up, 15 and 30 min after wake up) found in this sample was lower than morning cortisol responses found in normal healthy participants [57]. Looking at the cortisol responses to stress there was no significant change from before stress (T5) to after stress (T6). This latter finding is in line with other research investigating the relationship between early adversity in both men and women, and cortisol responses to stress [29]. Thus, the present study demonstrated blunted cortisol responses in a sample of forensic inpatients. Importantly, the correlation analyses revealed no relationships between cortisol responses and HF-HRV pre-intervention. However, post-intervention there were positive relationships between baseline HF-HRV and T2, T3, and T4, (i.e., the CAR). Thus, the relationship between cortisol responses and HF-HRV in this sample of forensic inpatients is different from what has been found in healthy adults without mental health problems [26]. However, no effects of vitamin D on cortisol responses were found nor did the correlational analyses show any relationships between vitamin D and cortisol responses.

The present study has several limitations that should be mentioned. One important limitation with this study is the small sample size. Unfortunately, it was not possible to recruit more participants. Over one-third of individuals screened were excluded, 18% of whom were excluded because they were already taking vitamin D supplements. As the present study included male forensic inpatients only, it is also difficult to say anything about the generalizability of the present results. Another important limitation with this study was that we did not measure serotonin before and after the experimental stress-induction procedures. For practical and economic reasons we were limited to one morning blood sample. Blood sampling before and after the experimental stress procedure might have been informative, but was not feasible. It should also be mentioned that cortisol studies in general are complicated to carry out. Given the limitations of the institution where the present study was carried out, morning saliva samples were collected when the staff could do the rounds on the living units. Investigating cortisol responses to stress is complicated because people respond differently to stressors as they perceive the stressors differently, and peaks in salivary cortisol levels may occur at multiple time points (varying from 5–20 min, see [36] for an overview). When it comes to cortisol studies, direct comparison with studies using other procedures should be interpreted cautiously. It is also important to keep in mind that subjects with “early adversity” do not constitute a homogenous group. Thus, similarities and differences between the studies may reflect multiple factors. In general one should always be aware of possible effects of other variables when it comes to intervention studies. With regard to the correlational analyses, we controlled for age, but the present study did not control for other potential variables such as personality traits or physical diseases. Moreover, the effect sizes for the psychophysiological results are small, so the results must be interpreted with caution.

Despite these limitations, the present study also has some strengths. It had a strong methodological design, including a double-blind randomized control trial of the effects of vitamin D on stress resilience, with careful monitoring of compliance with the intervention. Importantly, the degree of compliance was high (97%). The significant changes in HF-HRV and HR from baseline to the stress-induction condition, and from stress to the recovery period, indicate our stress-induction procedures were successful. Moreover, not many studies have investigated the diurnal pattern of cortisol responses in inpatients with complex mental health problems such as antisocial behavior problems. Most studies have looked at single-point determinants of cortisol levels, i.e., saliva or blood collected at just one time [30]. This might be due to the complexities regarding data collection. Collecting samples as investigated in the present study poses a substantial logistical challenge. Importantly, this study investigated the effects of vitamin D supplements in relation to the diurnal pattern, including a CAR in addition to cortisol responses to stress. Another important strength with this study is that it included multiple biological measures of stress resilience.

## 5. Conclusions

Overall, in a group of forensic inpatients the present study indicates that there are seasonal variations in resilience to stress. Resilience to stress seems to vary with seasonal changes in vitamin D level. Importantly, vitamin D supplementations during winter seems to influence resilience to stress during spring. However, future research should investigate the effects of vitamin D supplements in relation to stress resilience in other populations as well.

## Figures and Tables

**Table 1:** Descriptive statistics of the participants such as psychiatric characteristics (i.e., diagnoses), scores on the Childhood Trauma Questionnaire-Short Form (CTQ-SF) and Impact of Event Scale-Revised (IES-R), as well as volume of noise during performance of experimental tasks for the Control group and Intervention group (25(OH)D). Number of participants (*N*) are shown in percent.

Descriptive Variables	Control (%)	*N*	Intervention (%)	*N*	Total *N*
Diagnoses		39		39	78
Personality disorders	36		40		
Substance abuse	23		15		
Depression	3		1		
PTSD	1		1		
ADHD	5		1		
Bipolar	4		1		
CTQ-SF					
Emotional abuse		38		37	75
None (score 5–8)	11		8		
Low (9–12)	9		4		
Moderate (13–15)	5		11		
Severe (>16)	25		27		
Physical abuse		38		38	76
None (5–7)	11		13		
Low (8–9)	4		4		
Moderate (10–12)	11		3		
Severe (>13)	25		30		
Sexual abuse		38		38	76
None (5)	12		14		
Low (6–7)	0		0		
Moderate (8–12)	8		4		
Severe (>18)	30		32		
Emotional neglect		39		38	77
None (5–9)	14		19		
Low (10–14)	17		9		
Moderate (15–17)	5		4		
Severe (>18)	14		17		
Physical neglect		39		39	78
None (5–7)	19		22		
Low (8–9)	8		9		
Moderate (10–12)	13		12		
Severe (>13)	10		8		
IES-R *					
Two categories		38		39	77
Under score 24	23		25		
Over score24	25		26		
Four categories		38		39	77
Under score 24	23		25		
Between score 24–32	10		6		
Between score 33–36	1		4		
Between score 37 **–88	14		16		
Volume of noise pre-test		39		39	78
100 db	26		28		
90 db	9		13		
80 db	15		9		
Volume of noise post-test		38		39	77
100 db	29		31		
90 db	13		8		
80 db	8		9		

Note: Variations in numbers of participants concerning Childhood Trauma Questionnaire-Short Form (CTQ-SF) and Impact of Event Scale-Revised (IES-R) were due to omitted items/missing data. * Cut off scores for IES-R based on total score: total score divided into two categories: No post-traumatic stress disorder (PTSD) symptoms: <24. PTSD is a clinical concern: >24. Total score divided into 4 categories: No PTSD symptoms: <24. Partial PTSD symptoms: 24–32. Probable PTSD symptoms: 33–36. Severe PTSD: 37–88. ** 37 or more: This is high enough to suppress the immune system’s functioning (even 10 years after an impact event) [38]. ADHD = Attention Deficit Hyperactivity Disorder.

**Table 2:** Means and bracketed standard deviations across all variables. *N* = number of participants.

Descriptive Variables	Control	*N*	Vitamin D	*N*
Age	49(11)	39	48(11)	39
IQ	91(14)	39	92(14)	39
BMI	31(7)	39	30(7)	39
CTQ-SF				
Emotional abuse	14.47(6.38)	38	15.43(5.89)	37
Physical abuse	13.47(6.34)	38	14.53(7.05)	38
Sexual abuse	14.34(6.97)	38	15.11(7.76)	38
Emotional neglect	13.23(5.90)	39	13.37(6.45)	38
Physical neglect	9.74(4.2)	39	9.33(4.42)	38
IES-R				
Intrusion	10.39(7.90)	38	11.51(8.23)	39
Avoidant	9.97(7.29)	39	11.38(7.40)	39
Hyperarousal	5.10(5.84)	39	5.85(5.95)	39
Total score	25.65(19.60)	38	28.74(19.45)	39
Medication				
Antidepressant	0.82(1.12)	39	0.54(0.91)	39
Cardiovascular	1.23(1.35)	39	1.31(1.42)	39
**Dependent Variables**	**Control**	***n***	**Vitamin D**	***n***
(25-OH)D (nmol/L)–Pre	59(21)	39	63(16)	39
(25-OH)D (nmol/L)–Post	47(16)	39	76(21)	30
Serotonin (ng/mL)–Pre	4.20(0.70)	32	4.41(0.77)	37
Serotonin (ng/mL)–Post	3.92(1.22)	32	4.10(1.16)	37
HF-HRV–Pre				
Baseline	5.13(1.50)	33	4.94(1.42)	35
Stress	4.61(1.49)	33	4.66(1.23)	35
Recovery	4.92(1.34)	33	5.03(1.36)	35
HF-HRV–Post				
Baseline	4.83(1.38)	33	4.88(1.38)	35
Stress	4.69(1.24)	33	4.65(1.24)	35
Recovery	4.70(1.31)	33	5.14(1.36)	35
HR–Pre				
Baseline	70.98(12.67)	33	74.19(9.20)	35
Stress	72.54(12.06)	33	75.86(8.40)	35
Recovery	70.34(11.33)	33	73.08(8.64)	35
HR - Post				
Baseline	70.43(10.76)	33	73.73(12.11)	35
Stress	71.35(10.55)	33	75.28(11.50)	35
Recovery	70.47(10.72	33	72.77(11.54)	35
Cortisol–Pre				
T1	2.52(1.46)	35	3.14(1.53)	36
T2	9.94(10.83)	35	8.48(4.45)	36
T3	11.58(12.31)	35	11.38(6.32)	36
T4	10.10(9.44)	35	10.71(6.04)	36
T5	5.42(3.58)	35	6.09(4.36)	36
T6	5.18(3.60)	35	6.24(4.02)	36
T7	2.97(2.44)	35	4.64(8.15)	36
Cortisol–Post				
T1	3.11(2.29)	35	2.56(1.02)	36
T2	10.14(9.62)	35	9.50(5.53)	36
T3	12.28(9.87)	35	11.74(6.37)	36
T4	10.86(9.27)	35	10.45(5.14)	36
T5	5.32(2.55)	35	6.36(3.15)	36
T6	4.26(2.05)	35	5.42(2.81)	36
T7	2.84(1.85)	35	3.51(2.61)	36

Note: Reported means and standard deviations for descriptive data are from pre-intervention and *t*-tests revealed no significant differences between the groups (all *p* > 0.23). Cortisol values reported in the table were not log transformed for comparison reasons. IQ = Intelligence Quotient. BMI = Body Mass Index. CTQ-SF = Childhood Trauma Questionnaire-Short Form. IES-R = Impact of Event Scale-Revised. HF-HRV-Pre = High-frequency Heart Rate Variability Pre intervention. HF-HRV–Post = High-frequency Heart Rate Variability Post intervention. HR–Pre = Heart Rate Pre intervention. HR-Post = Heart Rate Post intervention.

**Table 3:** Shows correlations between vitamin D and biological markers of stress responses pre- and post-intervention.

**Biological Markers**		**Vitamin D**				**Vitamin D**		
	Pre-intervention		Post-intervention		Pre-intervention		Post-intervention	
	Zero-order correlations				Partial correlations controlling for age			
	r	*p*	r	*p*	r	*p*	r	*p*
HF-HRV Baseline	0.09	0.471	0.14	0.229	0.10	0.397	0.16	0.170
HF-HRV Stress	0.06	0.596	0.13	0.269	0.11	0.371	0.15	0.213
HF-HRV Recovery	0.02	0.887	0.19	0.112	0.03	0.810	0.21	0.067
HR Baseline	−0.04	0.773	−0.09	0.451	−0.04	0.722	−0.09	0.441
HR Stress	0.10	0.428	0.15	0.206	−0.04	0.752	−0.01	0.910
HR Recovery	−0.09	0.443	−0.06	0.603	−0.09	0.439	−0.06	0.588
Serotonin	0.27	0.025 *	0.23	0.046 *				
Cortisol T1	−0.00	0.995	−0.17	0.136				
Cortisol T2	0.00	0.989	−0.04	0.739				
Cortisol T3	0.07	0.532	0.05	0.674				
Cortisol T4	0.06	0.581	0.07	0.541				
Cortsiol T5	0.09	0.431	0.00	0.993				
Cortisol T6	−0.09	0.459	0.04	0.716				
Cortisol T7	0.05	0.654	0.05	0.664				

Note: HF-HRV = High-frequency Heart Rate Variability. HR = Hear Rate. * *p* < 0.05.

## Supplementary Materials

The following are available online at https://www.mdpi.com/2072-6643/12/11/3258/s1, Noise.
